# A Phase 2b Trial Evaluating the Safety, Tolerability, and Immunogenicity of a 6-Valent Group B *Streptococcus* Vaccine Administered Concomitantly With Tetanus, Diphtheria, and Acellular Pertussis Vaccine in Healthy Nonpregnant Female Individuals

**DOI:** 10.1093/infdis/jiaf096

**Published:** 2025-02-26

**Authors:** William B Smith, William Seger, Richard Chawana, Zahra Skogeby, Natalie C Silmon de Monerri, Ye Feng, Michelle Gaylord, Babalwa Jongihlati, Johannes Beeslaar, Julie M Skinner, Kara Bickham, Annaliesa S Anderson

**Affiliations:** Alliance for Multispecialty Research, Knoxville, Tennessee, USA; Avacare, Fort Worth, Texas, USA; Vaccine Clinical Research and Development Pfizer Inc, Johannesburg, Gauteng, South Africa; Vaccine Clinical Research and Development Pfizer Ltd, Marlow, United Kingdom; Vaccine Research and Development, Pfizer, Pearl River, New York, USA; Vaccine Research and Development, Pfizer, Pearl River, New York, USA; Vaccine Research and Development, Pfizer, Pearl River, New York, USA; Vaccine Research and Development, Pfizer, Pearl River, New York, USA; Vaccine Clinical Research and Development Pfizer Ltd, Marlow, United Kingdom; Vaccine Clinical Research and Development Pfizer Ltd, Marlow, United Kingdom; Vaccine Research and Development, Pfizer, Pearl River, New York, USA; Vaccine Research and Development, Pfizer, Pearl River, New York, USA

**Keywords:** group B *Streptococcus*, vaccines, safety, immunogenicity, Tdap vaccines co-administration

## Abstract

**Background:**

Maternal group B *Streptococcus* (GBS) infection is associated with substantial risk of preterm birth and infant mortality. Preventive approaches to protect infants from GBS infection are needed.

**Methods:**

In this phase 2b randomized study, healthy nonpregnant 18- to 49-year-old females were randomized 1:1:1 to receive the investigational 6-valent GBS polysaccharide conjugate vaccine (GBS6) and concomitant tetanus, diphtheria, and acellular pertussis vaccine (Tdap) (GBS6 + Tdap), GBS6 and placebo (GBS6 + placebo), or Tdap and placebo (Tdap + placebo). Primary safety endpoints included reactogenicity events within 7 days and adverse events (AEs) through 1 month after vaccination. Primary immunogenicity objectives were to describe immune responses induced by GBS6 + Tdap versus Tdap + placebo and versus GBS6 + placebo for pertussis, tetanus, and diphtheria Tdap antigens and the 6 GBS6 antigens.

**Results:**

Overall, 304 participants received study vaccination. Most reactogenicity events were mild or moderate in severity and balanced across vaccine groups. Frequency of AEs was ≤8.1% across vaccine groups. One month after vaccination, the proportion of participants achieving antibody concentrations ≥0.1 IU/mL for tetanus and diphtheria antigens was 100% in both the GBS6 + Tdap and Tdap + placebo groups. Immune responses to pertussis antigens were lower in the GBS6 + Tdap group compared to the Tdap + placebo group, with geometric mean ratios <0.6. No consistent effect on immune responses against each of the GBS6 serotypes after concomitant administration with Tdap was observed.

**Conclusions:**

GBS6 and Tdap administered concomitantly and alone were safe and well tolerated in healthy nonpregnant individuals. Similar immune responses were observed for Tdap when administered with GBS6 or when administered alone. These results will likely inform future studies in pregnant individuals.

**Clinical Trials Registration.** NCT04766086.

## Teaser

In this study in nonpregnant females, the investigational 6-valent group B *Streptococcus* polysaccharide conjugate vaccine (GBS6) and Tdap administered concomitantly and alone were safe and well tolerated and elicited immune responses to all antigens tested, although pertussis responses were lower with concomitant administration.

Group B *Streptococcus* (GBS) is a gram-positive bacterium that can cause serious disease such as sepsis, pneumonia, and, less frequently, meningitis [1]. GBS infection of the maternal urinary tract is associated with chorioamnionitis and can cause preterm labor, miscarriage, and stillbirth [2, 3]. Annually, invasive GBS disease is associated with approximately 392 000 cases, 91 000 infant deaths, and 46 000 stillbirths [4]. Additionally, up to 3.5 million preterm births annually are thought to be attributable to GBS [5].

The primary risk factor for early-onset GBS disease in neonates (characterized as infection within 7 days of birth) is maternal rectovaginal GBS colonization [1]. Global prevalence of rectovaginal GBS colonization in pregnant individuals is about 18%, although there is considerable regional variation (11%–35%) [6]. A recent meta-analysis estimated that 19.7 million pregnant individuals in 2020 had maternal GBS colonization, with approximately 231 800 neonates developing early-onset invasive GBS disease and 162 200 infants developing late-onset invasive GBS disease (7‒89 days after birth) [7].

Prophylactic antibiotic treatment from onset of labor is effective at preventing early-onset GBS disease in infants born to individuals with positive GBS cultures following screening between 35 and 37 weeks of each pregnancy, since colonization in individuals can vary over time, or in those who have other risk factors for intrapartum GBS colonization [1, 8, 9], including a previous infant with GBS disease, preterm labor, prolonged rupture of membranes >18 hours, age ≥35 years, and diabetes mellitus [10, 11]. Limitations with intrapartum antibiotic prophylaxis (IAP) include potential availability issues and variable implementation policies, potential contribution to antimicrobial resistance (ie, non-GBS pathogens, including *Escherichia coli*), and potential adverse effects such as disruption of the microbiome [12–14]. IAP also does not prevent GBS-associated stillbirth or maternal peripartum infections and is not effective against late-onset GBS disease in infants [13, 15].

Maternal vaccination against GBS during pregnancy has the potential to address shortcomings of IAP and reduce the burden of GBS disease in infants [13, 15]. GBS is an encapsulated bacterium with 10 defined capsular polysaccharide (CPS) serotypes [16]. Six serotypes (Ia, Ib, II, III, IV, and V) cause >95% of disease globally, with variability in their global prevalence and virulence [16–18]. CPSs are important bacterial virulence factors because they facilitate protection from host immune cells [19, 20]. Because CPSs are critical to the virulence of GBS, and anti-CPS antibodies can provide serotype-specific protection [16], multivalent glycoconjugate vaccines are in development for prevention of GBS-associated disease. Multivalent pneumococcal conjugate vaccines (PCVs) targeting CPS have had notable success in the reduction of pneumococcal disease burden worldwide, supporting application of the same technology for GBS vaccines [20, 21]. PCVs are safe and recommended in pregnant women who have risk factors for severe pneumococcal disease [22].

A maternal 6-valent CPS‒cross-reactive material 197 (CRM_197_) glycoconjugate vaccine (GBS6) targeting serotypes Ia, Ib, II, III, IV, and V to prevent invasive GBS disease in infants is currently in development [15, 17]. In a phase 1/2, dose-ranging study in 18- to 49-year-old nonpregnant adults, all GBS6 doses were well tolerated and elicited robust serotype-specific anti-CPS immunoglobulin G (IgG) responses that persisted for 6 months after vaccination [13]. In a phase 2 study in pregnant participants, GBS6 elicited immune responses to all 6 vaccine serotypes, which were then transferred to their infants at levels associated with reduced risk of invasive GBS disease through natural immunity [15].

GBS6 is being evaluated for administration within the same gestational timeframe as several other vaccines used during pregnancy, including the tetanus, diphtheria, and acellular pertussis vaccine (Tdap). Tdap is widely recommended for use in pregnant individuals for prevention of infant pertussis and neonatal tetanus [23, 24]. The recommended timeframe for Tdap administration during pregnancy varies between countries and regions; recommendations include 27–36 weeks’ gestation in the United States, Latin America, and the Caribbean, 16–32 weeks’ gestation in the United Kingdom, 27–32 weeks’ gestation in Canada, and mid-second and early third trimesters in Australia [23, 25–28]. Because maternal vaccinations are likely to be administered concomitantly, it is important to understand if coadministration of Tdap and GBS6 results in immune interference.

This phase 2b study assessed the safety, tolerability, and immunogenicity of GBS6 administered concomitantly with Tdap in healthy nonpregnant adult female individuals. These data will inform the design of subsequent studies in pregnant individuals and immunization programs potentially using these vaccines in the future.

## METHODS

### Study Design and Participants

This was a phase 2b, placebo-controlled, randomized, observer-blinded study conducted at 11 sites in the United States (NCT04766086). Healthy nonpregnant female participants 18–49 years of age were eligible to enroll. Key exclusion criteria included participants of childbearing potential who were pregnant, breastfeeding, or had a positive pregnancy urine test before vaccination and participants who were immunocompromised or had a history of invasive GBS disease. Full exclusion criteria, including prior or concomitant therapies, are outlined in the Supplementary Appendix.

### Study Vaccine and Procedures

Participants were randomized 1:1:1 to receive either 120 µg GBS6 and concomitant Tdap (GBS6 + Tdap), 120 µg GBS6 and placebo (GBS6 + placebo), or Tdap and placebo (Tdap + placebo). GBS6 was composed of multiple serotype (Ia, Ib, II, III, IV, and V) CPSs at 1 dose level (20 µg CPS/serotype/dose per vaccine dose of 120 µg) individually conjugated to CRM_197_ carrier protein. Tdap was a commercially available vaccine (Adacel, Sanofi Pasteur, Swiftwater, Pennsylvania) and placebo consisted of normal saline. GBS6, Tdap, and placebo were each administered as single 0.5-mL doses into the deltoid muscle.

The study was observer blinded, and the dosage form and presentation of GBS6, Tdap, and placebo were not matched. Staff preparing, dispensing, and administering vaccines were unblinded; all other study personnel and participants were blinded.

### Ethical Conduct

The study was conducted in accordance with the protocol, consensus ethical principles from international guidelines, including the Declaration of Helsinki and Council for International Organizations of Medical Sciences International Ethical Guidelines, applicable International Council on Harmonisation Guideline for Good Clinical Practice Guidelines, and other applicable laws and regulations, including privacy laws. The protocol was approved by the institutional review board before study initiation. Participants signed a statement of informed consent before enrollment.

### Objectives and Endpoints

#### Safety

The primary safety objective was to describe the safety and tolerability of GBS6 when concomitantly administered with Tdap (GBS6 + Tdap) or alone (GBS6 + placebo). Safety endpoints included local reactions (redness, swelling, and injection-site pain) and systemic events (fever, nausea/vomiting, diarrhea, headache, fatigue, muscle pain, and joint pain) within 7 days after vaccination, adverse events (AEs) through 1 month after vaccination, and medically attended AEs (MAEs) and serious AEs (SAEs) through 6 months after vaccination. Local reactions, systemic events, and use of antipyretic/pain medications were recorded by participants using an electronic diary and graded by severity (Supplementary Table 1).

#### Immunogenicity

The first primary immunogenicity objective was to describe immune responses induced by GBS6 + Tdap compared with Tdap + placebo. Immunogenicity endpoints for this objective included the difference in proportions of participants with anti‒tetanus toxoid (anti-TTd) antibody concentrations or anti‒diphtheria toxoid (anti-DTd) antibody concentrations ≥0.1 IU/mL for GBS6 + Tdap compared with Tdap + placebo 1 month after vaccination, and geometric mean ratios (GMRs) of anti‒pertussis toxin (anti-PT), anti‒filamentous hemagglutinin (anti-FHA), anti-pertactin (anti-PRN), and anti-fimbriae (anti-FIM; exploratory endpoint) antibodies for GBS6 + Tdap compared with Tdap + placebo 1 month after vaccination. The second primary immunogenicity objective was to describe immune responses induced by GBS6 + Tdap compared with GBS6 + placebo. Immunogenicity endpoints for this objective were GMRs of GBS serotype-specific anti-CPS IgG antibodies for GBS6 + Tdap compared with GBS6 + placebo 1 month after vaccination. The GMRs were derived from geometric mean concentrations (GMCs) of antibodies measured in the coadministration group compared with the non-coadministration group before and 1 month after vaccination. Any GMC levels above the lower limit of quantitation (LLOQ) were reported.

Blood samples for immunogenicity assessments were collected before vaccination and 1 month after vaccination. Concentrations of serum serotype‒specific anti-CPS IgG antibodies to Tdap antigens were determined using a multiplexed Luminex bead‒based DTP-6 IgG assay, and concentrations of serum serotype‒specific anti-CPS IgG antibodies specific for the 6 CPS serotypes in GBS6 were determined using a validated multiplex direct Luminex-based immunoassay [29].

### Statistical Analysis

Formal statistical hypotheses were not assessed in this descriptive study. Binary variables were reported as percentages with exact 95% confidence intervals (CIs) computed using the Clopper and Pearson method.

Local reactions, systemic events, and AEs were assessed in the safety population, which included participants who received ≥1 dose of study vaccine and had ≥1 safety assessment after vaccination.

Immunogenicity was assessed in the immunogenicity population, which consisted of participants who were eligible, received all doses of study vaccine according to which they were randomized, had blood drawn for immunogenicity testing within the specified timeframe for 1 month after vaccination, had ≥1 valid and determinate assay result, and had no major protocol violations. The GMCs of antibodies and associated 2-sided 95% CIs were derived by calculating group means and CIs on the natural log scale based on the Student *t* distribution and then exponentiating the results. Concentrations below the LLOQ were set to 0.5 × LLOQ for GMC analysis. Geometric mean fold rises were calculated as the mean difference of an individual participant's logarithmically transformed antibody levels (postvaccination minus baseline) and transformed back to the original units. The 95% CI was computed by back-transformation of the 95% CI using the Student *t* distribution for the mean difference of measures on the logarithmically transformed assay results. GMRs were calculated as the group mean difference of logarithmically transformed antibody levels, transformed back to the original units. Two-sided 95% CIs were calculated using the Student *t* distribution for the mean difference of measures on the logarithmically transformed assay results, with confidence limits transformed back to the original units.

## RESULTS

### Participants

**Figure 1.**
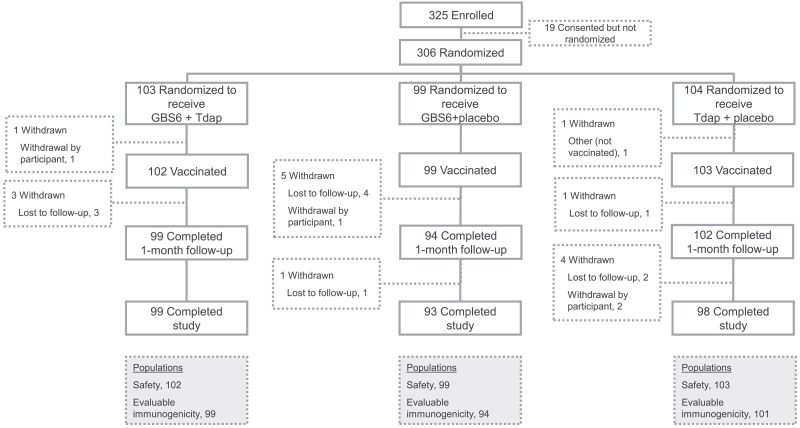
Participant disposition. Participants who completed the 1-month postvaccination follow-up visit (per protocol amendment 2) or who completed the 6-month postvaccination visited (per protocol amendment 3) were considered to have completed the study. Of the 19 participants who were consented but not randomized, 18 did not pass the screening and 1 was not randomized because randomization closed during the screening visit. GBS6 indicates group B *Streptococcus* 6-valent polysaccharide conjugate vaccine; Tdap indicates tetanus, diphtheria, and acellular pertussis vaccine.

From 12 August 2022, to 27 April 2023, 306 participants were randomized and 304 were vaccinated and included in the safety population; of these, 102 received GBS6 + Tdap, 99 received GBS6 + placebo, and 103 received Tdap + placebo. Overall, 96.1%, 93.9%, and 94.2% of participants who received GBS6 + Tdap, GBS6 + placebo, and Tdap + placebo, respectively, completed the study (Figure 1). Participant demographics are shown in Table 1 and were generally well balanced across groups. The median age overall was 36.0 (range, 18‒49) years and 73.4% of participants were White.

**Table 1.** Demographic and Baseline Characteristics by Vaccine Group (Safety Population)

Characteristic	GBS6 + Tdap (n = 102)	GBS6 + Placebo (n = 99)	Tdap + Placebo (n = 103)	Total (n = 304)
Sex				
Female	102 (100.0)	99 (100.0)	103 (100.0)	304 (100.0)
Race				
White	80 (78.4)	68 (68.7)	75 (72.8)	223 (73.4)
Black or African American	18 (17.6)	19 (19.2)	19 (18.4)	56 (18.4)
Asian	3 (2.9)	5 (5.1)	1 (1.0)	9 (3.0)
Multiracial	0	4 (4.0)	4 (3.9)	8 (2.6)
American Indian or Alaska Native	1 (1.0)	3 (3.0)	0	4 (1.3)
Not reported	0	0	4 (3.9)	4 (1.3)
Ethnicity				
Non-Hispanic/Non-Latino	93 (91.2)	86 (86.9)	86 (83.5)	265 (87.2)
Hispanic or Latino	8 (7.8)	12 (12.1)	16 (15.5)	36 (11.8)
Not reported	1 (1.0)	1 (1.0)	1 (1.0)	3 (1.0)
Age at vaccination, y				
Mean (SD)	35.4 (8.54)	34.8 (8.60)	35.5 (8.59)	35.3 (8.55)
Median (min, max)	35.5 (18, 49)	36.0 (20, 49)	36.0 (18, 49)	36.0 (18, 49)

Data are n (%) unless noted otherwise.

GBS6 indicates group B *Streptococcus* 6-valent polysaccharide conjugate vaccine; SD indicates standard deviation; Tdap indicates tetanus, diphtheria, and acellular pertussis vaccine.

### Safety

**Figure 2.**
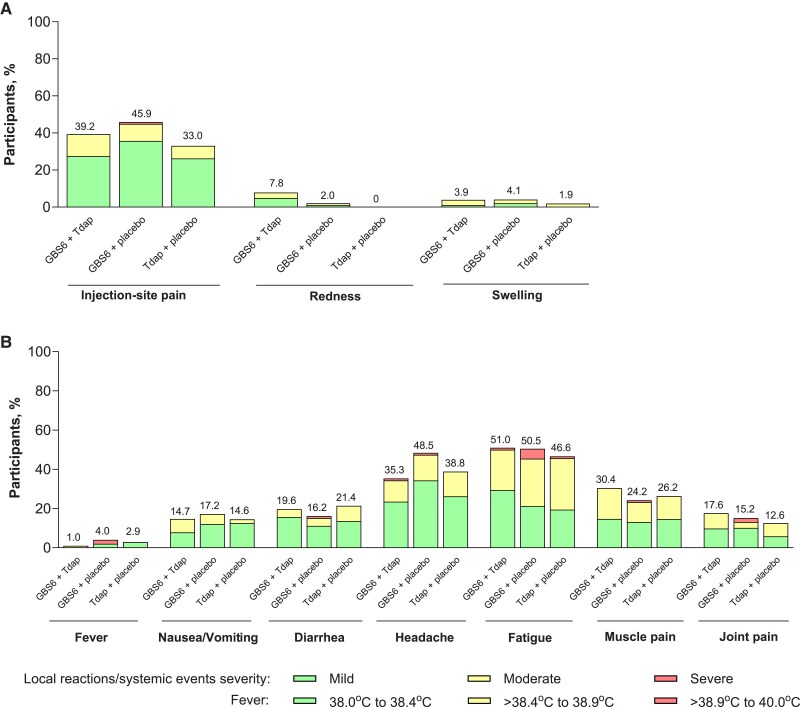
Local reactions (*A*) and systemic events (*B*) by maximum severity across vaccination groups within 7 days after vaccination (safety population). Local reactions are those at the injection site of the left deltoid muscle (ie, site of vaccination for GBS6 for the GBS6 + Tdap and GBS6 + placebo groups and for placebo in the Tdap + placebo group). GBS6 indicates group B *Streptococcus* 6-valent polysaccharide conjugate vaccine; Tdap indicates tetanus, diphtheria, and acellular pertussis vaccine.

Local reactions and systemic events were mostly mild or moderate in severity with no clear differences in frequencies between the 3 vaccination groups (Figure 2). The most frequently reported local reaction was injection-site pain (GBS6 + Tdap, 39.2%; GBS6 + placebo, 45.9%; Tdap + placebo, 33.0%; Figure 2*A*). One participant in the GBS6 + placebo group reported severe injection-site pain; all other local reactions were mild or moderate and no grade 4 events were reported. In any vaccine group, the median onset day of any local reaction was the same day as the day of vaccination, and all local reactions lasted for a median of ≤2.5 days, except for swelling in the GBS6 + Tdap group, which lasted for a median of 4.5 days.

The most frequently reported systemic event was fatigue (GBS6 + Tdap, 51.0%; GBS6 + placebo, 50.5%; Tdap + placebo, 46.6%), followed by headache (GBS6 + Tdap, 35.3%; GBS6 + placebo, 48.5%; Tdap + placebo, 38.8%; Figure 2*B*). Fever >38.9°C was reported in 2 participants (2.0%) in the GBS6 + placebo group. All systemic events lasted for a median of ≤3.0 days across all vaccine groups. Fatigue had a median onset of 1.0 days after vaccination in the Tdap + placebo group and 1.5 days after vaccination in both the GBS6 + Tdap and GBS6 + placebo groups; all other systemic events had a median onset of 2.0–3.0 days after vaccination. Antipyretic/pain medications were used for a median of 2.0 days.

Adverse events through 1 month after vaccination are summarized in Supplementary Table 2. Six (5.9%), 8 (8.1%), and 4 (3.9%) participants reported AEs in the GBS6 + Tdap, GBS6 + placebo, and Tdap + placebo groups, respectively. The most common AEs were cough (GBS6 + Tdap, 2 participants [2.0%]) and COVID-19 (GBS6 + placebo, 2 participants [2.0%]).

Adverse events assessed by the investigator as related to vaccination were reported by 1 participant in the GBS6 + Tdap group (axillary pain) and 1 in the GBS6 + placebo group (lymphadenopathy); no AEs led to withdrawal from the study. One SAE was reported (bile duct stone in a participant in the Tdap + placebo group who had a history of gallstones), which was assessed as not related to the vaccine. Twenty-four MAEs were reported in 5 (4.9%), 4 (4.0%), and 7 (6.8%) participants in the GBS6 + Tdap, GBS6 + placebo, and Tdap + placebo groups, respectively; none were assessed as related to vaccination. Six MAEs occurred within 1 month of vaccination, and 18 occurred 1–6 months after vaccination.

### Immunogenicity

No major protocol violations were reported among study participants; 294 participants were included in the evaluable immunogenicity population for analyses, with 10 participants excluded because of missing protocol-defined assay results.

#### Immune Responses to Tdap

Before vaccination, the percentage of participants with anti-TTd antibody concentrations ≥0.1 IU/mL was 99.0% in both the GBS6 + Tdap and Tdap + placebo groups; 1 month after vaccination, the corresponding percentages increased to 100% in both groups (between-group difference, 0%; Supplementary Figure 1*A*). Similarly, before vaccination, 89.9% and 88.0% of participants had anti-DTd antibodies at concentrations ≥0.1 IU/mL in the GBS6 + Tdap and Tdap + placebo groups, respectively, and 1 month after vaccination, the corresponding percentages increased to 100% in both groups (between-group difference, 0%; Supplementary Figure 1*A*). Thus, for these primary endpoints, no differences in the percentages of participants reaching these thresholds were observed.

**Figure 3.**
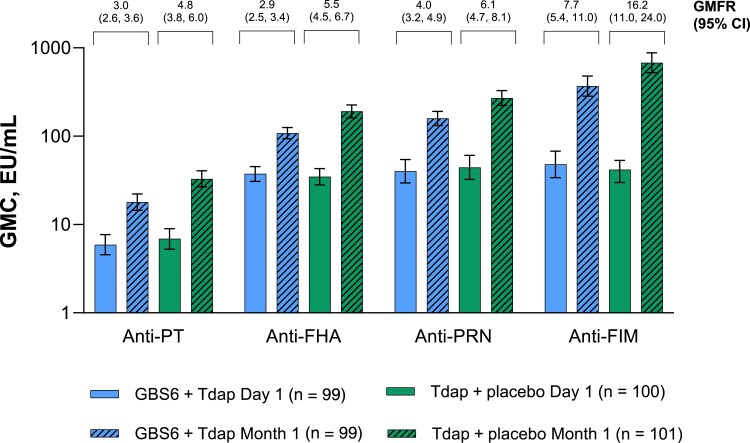
Geometric mean concentrations (GMCs) and geometric mean fold rises (GMFRs) from before to 1 month after vaccination of anti-pertussis components. Day 1 is before vaccination. Lower limit of quantitation values were anti–pertussis toxin (PT) = 0.9, anti–filamentous hemagglutinin (FHA) = 2.9, anti-pertactin (PRN) = 3, and anti-fimbriae (FIM) = 10.6. Error bars are the 95% confidence intervals (CIs), which are back-transformations of CIs based on the Student *t* distribution for the mean logarithm of the concentrations. The 95% CIs for GMFRs are back-transformations of CIs based on the Student *t* distribution for the mean fold rise. GBS6 indicates group B *Streptococcus* 6-valent polysaccharide conjugate vaccine; Tdap indicates tetanus, diphtheria, and acellular pertussis vaccine.

Both GBS6 + Tdap and Tdap + placebo elicited responses to all pertussis components 1 month after vaccination (Figure 3). Although not powered to assess differences between groups, coadministration of GBS6 + Tdap resulted in lower GMCs to all pertussis antigens compared with Tdap alone. GMRs for anti-PT, anti-FHA, anti-PRN, and anti-FIM antibodies in the GBS6 + Tdap group to Tdap + placebo 1 month after vaccination were 0.546, 0.567, 0.588, and 0.545, respectively (Supplementary Figure 1*B*).

#### Immune Responses to GBS6

**Figure 4.**
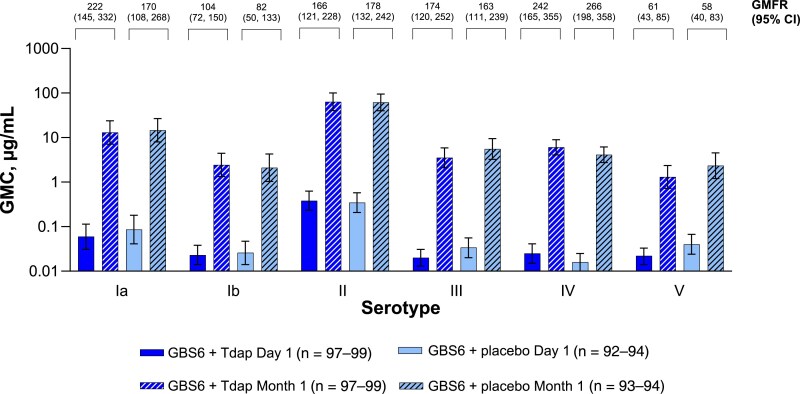
Group B *Streptococcus* serotype-specific anti–capsular polysaccharide immunoglobulin G geometric mean concentrations (GMCs) and geometric mean fold rises (GMFRs) from before to 1 month after vaccination. Day 1 is before vaccination. Lower limit of quantitation (LLOQ) values were serotype Ia = 0.002, Ib = 0.005, II = 0.022, III = 0.009, IV = 0.004, and V = 0.010. Assay results below the LLOQ were set to 0.5 × LLOQ. Error bars are the 95% confidence intervals (CIs), which are back-transformations of CIs based on the Student *t* distribution for the mean logarithm of the concentrations. The 95% CIs for GMFRs are back-transformations of CIs based on the Student *t* distribution for the mean fold rise. GBS6 indicates group B *Streptococcus* 6-valent polysaccharide conjugate vaccine; Tdap indicates tetanus, diphtheria, and acellular pertussis vaccine.

**Figure 5.**
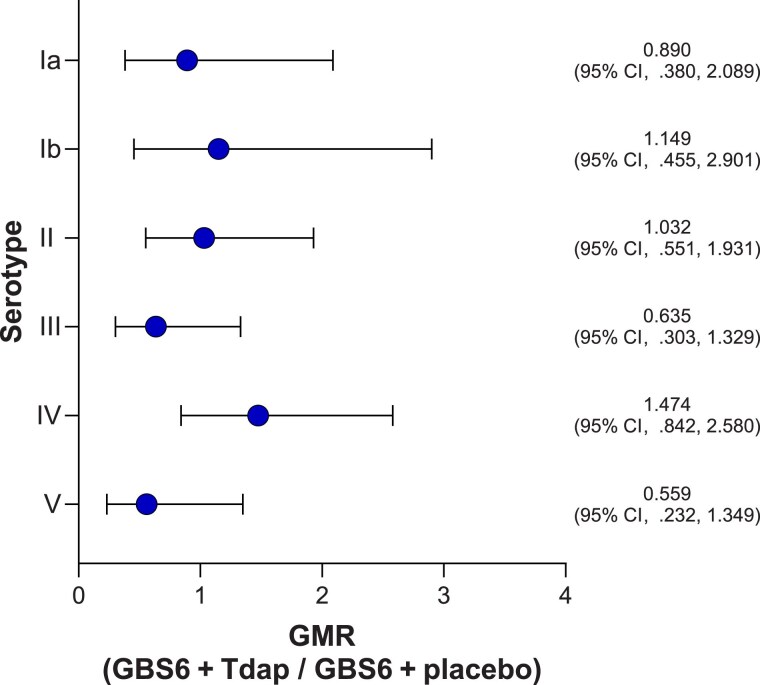
Group B *Streptococcus* serotype-specific anti–capsular polysaccharide immunoglobulin G geometric mean ratios (GMRs) (group B *Streptococcus* 6-valent polysaccharide conjugate vaccine [GBS6] + tetanus, diphtheria, and acellular pertussis vaccine [Tdap]/GBS6 + placebo) at 1 month after vaccination (evaluable immunogenicity population). GMRs (GBS6 + Tdap/GBS6 + placebo) were calculated by back-transforming the mean differences between the 2 groups on the logarithmic scale. Error bars are the 95% confidence intervals (CIs), which are back-transformations of CIs based on the Student *t* distribution for the mean logarithm of the concentrations. N values are 97–99 and 93–94 for the GBS6 + Tdap and GBS6 + placebo groups, respectively.

Compared with levels before vaccination, serotype-specific anti-CPS IgG GMCs for all GBS serotypes increased 1 month after vaccination in both the GBS6 + Tdap and GBS6 + placebo groups (Figure 4). The impact of concomitant administration was variable across serotypes, but the wide CIs observed complicate interpretation. GBS serotype-specific anti-CPS IgG GMRs (95% CIs) at 1 month after vaccination were <1 for serotypes Ia (0.890 [.380–2.089]), III (0.635 [.303–1.329]), and V (0.559 [.232–1.349]) and >1 for serotypes Ib (1.149 [.455–2.901]), II (1.032 [.551–1.931]), and IV (1.474 [.842–2.580]; Figure 5).

## DISCUSSION

The 6-valent GBS6 vaccine, currently under development as a maternal vaccine to prevent invasive GBS disease in young infants, was evaluated for safety and potential immune interference when concomitantly administered with Tdap in nonpregnant females. These findings support that GBS6 concomitantly administered with Tdap was well tolerated and had an acceptable safety profile. These safety findings are consistent with those from a previous study wherein GBS6 was administered as a single 20-µg/CPS/serotype dose to nonpregnant adults [13]. In both studies, the safety profile of GBS6 was characterized primarily by mild injection-site pain and mild and moderate systemic events; however, rates of unsolicited AEs were lower in the current study (3.9%‒8.1% of participants) compared with 40% reported in the previous study. The reasons for the differences in AE rates between studies are unclear. The safety profile in the current study was also consistent with safety profiles reported in previous studies in which Tdap was concomitantly administered with other vaccines including the respiratory syncytial virus prefusion F vaccine (RSVpreF), meningococcal conjugate vaccines, and the 13-valent pneumococcal CRM_197_ conjugate vaccine in healthy nonpregnant individuals [30–33].

The immune responses induced by Tdap when administered concomitantly with GBS6, as determined by the proportion of participants achieving antibody concentrations ≥0.1 IU/mL, were similar to the responses induced by Tdap alone for tetanus and diphtheria antigens. Immune responses to pertussis antigens were lower with coadministration of GBS6. The clinical relevance of lower immune responses to pertussis antigens is not fully understood since a correlate of protection for pertussis vaccines has not yet been established [34], and notably, the current study was not powered to evaluate differences in immunogenicity between study arms. Similar attenuated responses to pertussis antigens after coadministration of Tdap with other vaccines, including influenza vaccines, meningococcal conjugate vaccines, RSVpreF, and adjuvanted recombinant zoster vaccine, have been reported in adolescent and adult populations [30, 31, 35–39].

No consistent effect on immune responses against each of the GBS6 serotypes after concomitant administration with Tdap was observed in the current study. Immune responses induced by GBS6 for serotypes Ia, III, and V when administered concomitantly with Tdap were lower compared with the responses induced by GBS6 alone, whereas coadministration of GBS6 and Tdap induced higher immune responses for GBS6 serotypes Ib, II, and IV compared with the immune responses induced by GBS6 alone. With no specific pattern of the effect of Tdap coadministration on the GBS6 antigens, the wide CIs observed, and the fact that a correlate of protection has not been established for GBS, the clinical relevance of these findings is unclear.

Although tetanus-diphtheria (Td) vaccines are recommended over the monovalent tetanus toxoid (TT) vaccine [40], many low-income regions, where GBS disease burden is highest, use TT instead of Tdap [3, 5]. The World Health Organization recommends that women receive 1 dose of TT during pregnancy and those who were not previously vaccinated against TT should receive 1 dose at their first antenatal visit and then 4 weeks later at the next antenatal care visit [41]. Therefore, administration of the monovalent TT vaccine could coincide with GBS vaccination. Although the current data show that coadministration of GBS6 with Tdap does not affect anti-TTd immune responses, it is unknown whether this would also be true for coadministration of GBS6 with the monovalent TT vaccine.

A limitation of this study is that it was not powered to assess noninferiority, precluding formal statistical hypothesis testing for immunogenicity endpoints. Future studies should ideally include sufficient participants to allow formal statistical analyses. The study was conducted in the United States and the population was mostly White, potentially impacting the generalizability of the findings across other populations and regions. Other limitations include conducting the study in nonpregnant rather than pregnant individuals, which prevented assessment of the potential influence of coadministration of Tdap with GBS6 on antibody levels in cord blood; however, large differences in immune responses to Tdap and GBS6 antigens between pregnant and nonpregnant populations are not anticipated.

## CONCLUSIONS

To our knowledge, this study is the first time a GBS polysaccharide conjugate vaccine has been concomitantly administered with Tdap. This study provided further support for the safety, tolerability, and immunogenicity of GBS6 vaccine and provided evidence supporting coadministration of GBS6 with Tdap. Taken together, these findings will help support further clinical development of GBS6 and hopefully ultimately inform essential technical committees on programmatic considerations for maternal immunizations.

## Supplementary Material

jiaf096_Supplementary_Data

## Supplementary Data

Supplementary materials are available at *The Journal of Infectious Diseases* online (http://jid.oxfordjournals.org/). Supplementary materials consist of data provided by the author that are published to benefit the reader. The posted materials are not copyedited. The contents of all supplementary data are the sole responsibility of the authors. Questions or messages regarding errors should be addressed to the author.

## Notes

***Author contributions.*** W. B. S. was involved in the concept and study design and data interpretation. W. S. was involved in data acquisition or generation. R. C. was involved in data interpretation, analysis, and verification. Z. S., N. C. S. M., B. J., J. M. S., K. B., and A. S. A. were involved with the concept and study design and data interpretation, analysis, and verification. Y. F. was involved with the concept and study design; data interpretation, analysis, and verification; and statistical analysis. M. G. and J. B. were involved in data interpretation, analysis, and verification. All authors critically reviewed the manuscript and approved the final draft.

***Acknowledgments.*** The authors thank those who participated in this study. Medical writing support was provided by John Teiber, PhD, and James Currie, PhD, of ICON (Blue Bell, Pennsylvania) and was funded by Pfizer Inc.

***Data sharing.*** Upon request, and subject to review, Pfizer will provide the data that support the findings of this study. Subject to certain criteria, conditions, and exceptions, Pfizer may also provide access to the related individual de-identified participant data. See https://www.pfizer.com/science/clinical-trials/trial-data-and-results for more information.

***Financial support.*** This study was sponsored by Pfizer Inc.
