# Supplementary material for: A Phase 2b Trial Evaluating the Safety, Tolerability, and Immunogenicity of a 6-Valent Group B Streptococcus Vaccine Administered Concomitantly With Tetanus, Diphtheria, and Acellular Pertussis Vaccine in Healthy Nonpregnant Female Individuals
Source: J Infect Dis. 2025 Feb 26;231(6):e1065–74. doi: 10.1093/infdis/jiaf096 (PMC12247796; doi:10.1093/infdis/jiaf096)
Supplement: jiaf096_Supplementary_Data [file jiaf096_supplementary_data.pdf]

SUPPLEMENTARY APPENDIX

Additional Eligibility Criteria

Healthy female individuals ≥18 and ≤49 years of age were eligible if they were not pregnant or breastfeeding and were not of childbearing potential or were of childbearing potential and using an acceptable contraceptive method during the intervention period (for a minimum of 28 days after the last dose of study intervention). A participant was considered fertile following menarche and until becoming postmenopausal unless permanently sterile. If fertility were unclear (eg, amenorrhea in adolescents or athletes) and a menstrual cycle could not be confirmed before the first dose of study intervention, additional evaluation should have been considered. The investigator evaluated the effectiveness of the contraceptive method in relationship to the first dose of study intervention. Participants were also included if they were willing and able to comply with scheduled visits, investigational plan, laboratory tests, lifestyle considerations, and other study procedures, including completion of the e-diary from Day 1 to Day 7 following administration of investigational product; were healthy at enrollment, as determined by medical history, physical examination, and clinical judgment of the investigator to be eligible for inclusion; were expected to be available for the duration of the study and could be contacted by telephone during study participation; and were capable of giving personal informed consent.

Participants were excluded if they were pregnant, breastfeeding, or had a positive urine pregnancy test at Visit 1 (before vaccination); as were individuals of childbearing potential who were, in the opinion of the investigator, sexually active and at risk for pregnancy; and those of childbearing potential unwilling or unable to use effective methods of contraception from the signing of the informed consent until ≥28 days after the last dose of investigational product. Individuals were also excluded if they had a history of severe adverse reaction associated with a vaccine and/or severe allergic reaction (eg, anaphylaxis) to any component of the investigational product or any diphtheria toxoid–containing or CRM_{197}-containing vaccine; had history of microbiologically proven invasive disease caused by group B streptococcus; were immunocompromised with known or suspected immunodeficiency; or had bleeding diathesis or a condition associated with prolonged bleeding that would in the opinion of the investigator contraindicated intramuscular injection. They were also excluded if they had other medical or psychiatric condition including recent (within the past year) or active suicidal ideation/behavior or laboratory abnormality that may have increased the risk of study participation or, in the investigator's judgment, made the participant inappropriate for the study; had received previous vaccination with any licensed or investigational GBS vaccine, or planned receipt during the participant's participation in the study (through the 6-month follow-up visit [Visit 3]); had received vaccination within 5 years with tetanus and diphtheria toxoids and acellular pertussis–containing vaccines (Tdap) before investigational product administration; or had received treatment with immunosuppressive therapy, including cytotoxic agents or systemic corticosteroids, eg, for cancer or an autoimmune disease, or planned receipt through the 1-month follow-up visit (Visit 2); were vaccinated with diphtheria- or CRM_{197}-containing vaccine(s) from 6 months before investigational product administration, or planned receipt through the 1-month follow-up visit; had received or planned to receive blood/plasma products or immunoglobulin from 60 days before investigational product administration through the 1-month follow-up visit; or were participating in other studies involving investigational drug(s) within 28 days before study entry and/or during study

participation. Participation in purely observational studies was acceptable. Investigator site staff or Pfizer employees directly involved in the conduct of the study, site staff otherwise supervised by the investigator, and their respective family members were also excluded.

Table S1. Severity Grading of Local Reactions and Systemic Events

Local Reaction or Systemic Event	Mild	Moderate	Severe	Grade 4
Local reaction				
Injection-site pain	Does not interfere with activity	Repeated use of nonnarcotic pain reliever >24 h or interferes with activity	Any use of narcotic pain reliever or prevents daily activity	Emergency department visit or hospitalization
Redness	>2.0–5.0 cm	>5.0–10.0 cm	>10.0 cm	Necrosis or exfoliative dermatitis
Swelling	>2.0–5.0 cm	>5.0–10.0 cm	>10.0 cm	Necrosis
Systemic event				
Nausea/vomiting	No interference with activity or 1–2 times in 24 h	Some interference with activity or >2 times in 24 h	Prevents daily activity; requires intravenous hydration	Emergency department visit or hospitalization for hypotensive shock
Diarrhea	2–3 loose stools in 24 h	4–5 loose stools in 24 h	≥6 loose stools in 24 h	Emergency department visit or hospitalization for severe diarrhea
Headache	No interference with activity	Repeated use of nonnarcotic pain reliever >24 hours or some interference with activity	Significant; any use of narcotic pain reliever or prevents daily activity	Emergency department visit or hospitalization for severe headache
Fatigue/tiredness	No interference with activity	Some interference with activity	Significant; prevents daily activity	Emergency department visit or hospitalization for severe fatigue
Muscle pain	No interference with activity	Some interference with activity	Significant; prevents daily activity	Emergency department visit or hospitalization for severe muscle pain
Joint pain	No interference with activity	Some interference with activity	Significant; prevents daily activity	Emergency department visit or hospitalization for severe joint pain

Table S2. Adverse Events Through 1 Month After Vaccination (Safety Population)

Event	GBS6+Tdap (N=102)	GBS6+Placebo (N=99)	Tdap+Placebo (N=103)
Any	6 (5.9)	8 (8.1)	4 (3.9)
Serious	0	0	1 (1.0)
Immediate	0	0	0
Severe	0	0	1 (1.0)
Related	1 (1.0)	1 (1.0)	0
Medically attended	0	3 (3.0)	3 (2.9)
Leading to withdrawal	0	0	0

All data are n (%).

GBS6=group B streptococcus 6-valent polysaccharide conjugate vaccine; Tdap=tetanus, diphtheria, and acellular pertussis vaccine.

Figure S1. (A) Difference in percentage of participants achieving anti-TTd and anti-DTd concentrations ≥0.1 IU/mL and (B) GMRs of pertussis antigen antibodies at 1 month after vaccination with GBS6+Tdap or Tdap+placebo (evaluable immunogenicity population)

In panel A, the risk difference was computed as the percentage of participants achieving anti-TTd or anti-DTd concentration ≥0.1 IU/mL at 1 month after vaccination for GBS6+Tdap minus the percentage of participants in the Tdap+placebo group. In panel B, the GMR (GBS6+Tdap / Tdap+placebo) was calculated by back transforming the mean difference between the 2 groups on the logarithmic scale. Error bars in panel A are the 2-sided 95% CIs based on the Miettinen and Nurminen method and error bars in panel B are the 2-sided 95% CIs that are back transformations of CIs based on the Student *t* distribution for the mean logarithm of the concentration. N values are 99 for the GB6+Tdap group and 101 for the Tdap+placebo group. DTd=diphtheria toxoid; FHA=filamentous hemagglutinin; FIM=fimbriae; GBS6=group B streptococcus 6-valent polysaccharide conjugate vaccine; GMR=geometric mean ratio; PRN=pertactin; PT=pertussis toxin; Tdap=tetanus, diphtheria, and acellular pertussis vaccine; TTd=tetanus toxoid.

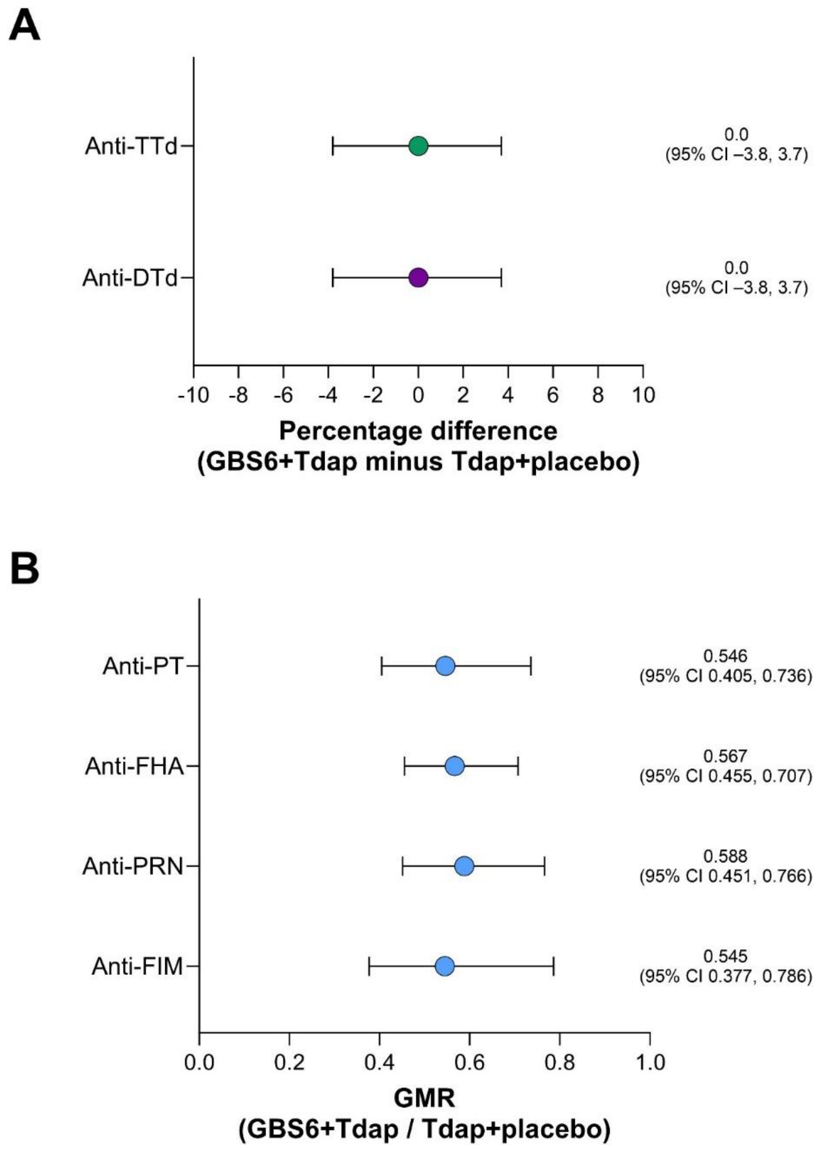
A
Anti-TTd
0.0
(95% CI –3.8, 3.7)
Anti-DTd
0.0
(95% CI –3.8, 3.7)
-10 -8 -6 -4 -2 0 2 4 6 8 10
Percentage difference
(GBS6+Tdap minus Tdap+placebo)
B
Anti-PT
0.546
(95% CI 0.405, 0.736)
Anti-FHA
0.567
(95% CI 0.455, 0.707)
Anti-PRN
0.588
(95% CI 0.451, 0.766)
Anti-FIM
0.545
(95% CI 0.377, 0.786)
0.0 0.2 0.4 0.6 0.8 1.0
GMR
(GBS6+Tdap / Tdap+placebo)
